# Frequently asked questions about the ISAPP postbiotic definition

**DOI:** 10.3389/fmicb.2023.1324565

**Published:** 2024-01-10

**Authors:** Gabriel Vinderola, Mary Ellen Sanders, Marla Cunningham, Colin Hill

**Affiliations:** ^1^Instituto de Lactología Industrial (CONICET-UNL), Faculty of Chemical Engineering, National University of Litoral, Santa Fe, Argentina; ^2^International Scientific Association for Probiotics and Prebiotics, Centennial, CO, United States; ^3^International Scientific Association for Probiotics and Prebiotics, Brisbane, QLD, Australia; ^4^APC Microbiome Ireland, University College Cork, Cork, Ireland

**Keywords:** postbiotics, International Scientific Association for Probiotics and Prebiotics, ISAPP, biotic, inanimate microorganism

## Abstract

The term postbiotic was defined by the International Scientific Association of Probiotics and Prebiotics (ISAPP) as “*a preparation of inanimate microorganisms and/or their components that confers a health benefit on the host*.” Although the ISAPP definition is widely cited, some concerns were aired after publication, and alternative definitions of postbiotic, as well as different terms for inactivated microbes, have been previously suggested. This paper addresses questions about the ISAPP definition that have been raised in different forums, including scientific meetings, social media commentary and personal communications. We focus on the rationale, scope, wording, composition and commercial implementation, as well as what is expected of postbiotics regarding safety, efficacy, quantification and mechanisms of action. We hope that exploring these questions will further clarify the definition and its scope and support a common understanding of the concept of postbiotics.

## 1 Introduction

In 2021 the International Scientific Association of Probiotics and Prebiotics (ISAPP) proposed the following definition for the term postbiotic; “*a preparation of inanimate microorganisms and/or their components that confers a health benefit on the host*” (Salminen et al., 2021a). This definition was the output from a consensus panel organized by ISAPP involving 11 participants from 10 countries with a diverse range of backgrounds spanning gastroenterology, pediatrics, metabolomics, microbiology, immunology, functional genomics, probiotic and host interactions as well as regulatory affairs. The resulting publication (Salminen et al., 2021a) has been widely accessed and cited. A published criticism of the definition raised concerns about alignment with previous definitions as well as queries on terminology (Aguilar-Toalá et al., 2021), to which a reply was published (Salminen et al., 2021b). The ISAPP definition has been further discussed in various forums, including panel discussions at scientific meetings, social media commentary and personal communications. This commentary responds to frequently asked questions (FAQs) about the ISAPP definition that are focused on the rationale, scope, wording, composition and commercial implementation, as well as what is expected of postbiotics regarding safety, efficacy, quantification and known mechanisms of action. We hope that exploring these FAQs will further clarify the definition and its scope and support a common understanding of the concept of postbiotics.

## 2 Why was a consensus definition for postbiotics needed?

With growing interest in the use of non-viable microbes and microbial metabolites as targeted interventions for human and animal health, we felt it would be useful to align the field around common terminology in referring to these substances. Without consensus on definitions and proper use of terms, we risk confusion across scientific, consumer and regulatory arenas, hampering the growth potential of this nascent and exciting field. We noted that six previous definitions of postbiotics had been published, but the panel concluded that individually and collectively they incorporated both limitations and contradictions, which were discussed by Salminen et al. (2021b) and Vinderola et al. (2022), and that a more precise definition for postbiotics would benefit the field.

## 3 Why does the ISAPP definition focus on non-viable cells or cell components rather than metabolites?

The ISAPP definition of postbiotics focuses on the beneficial role of inanimate microbes and their component structures. Derived from the Greek language, the prefix ‘*post*’ means ‘after’ and the word ‘*biotic*’ means ‘living things’, indicating that a postbiotic should refer to something that was living and is now ‘after life’, or inanimate. Please see question 8 for a detailed discussion about the use of the term inanimate. Microbial metabolites, such as vitamins or short chain fatty acids, are not living things and so cannot have an ‘after-life.’ For that reason, compositions consisting only of metabolites were excluded from the postbiotic concept. Existing nomenclature can be used in the case of simple metabolites, such as butyrate, or collective names such as cell-free filtrate can be used for more complex preparations not containing inanimate microbes or microbial components. This approach prevents the injudicious situation where a microbe-derived metabolite or metabolite mixture is called a ‘postbiotic’ but an identical chemically synthesized preparation is not. Some authors have proposed the use of the term ‘metabiotics’ to collectively refer to metabolites able to confer a health benefit (Sadeghi et al., 2023; Tenea, 2023). However, we take the view that before introducing a new term and its definition, a careful and thorough expert debate that can precisely differentiate any new terminology from existing definition be undertaken.

**Figure 1:**
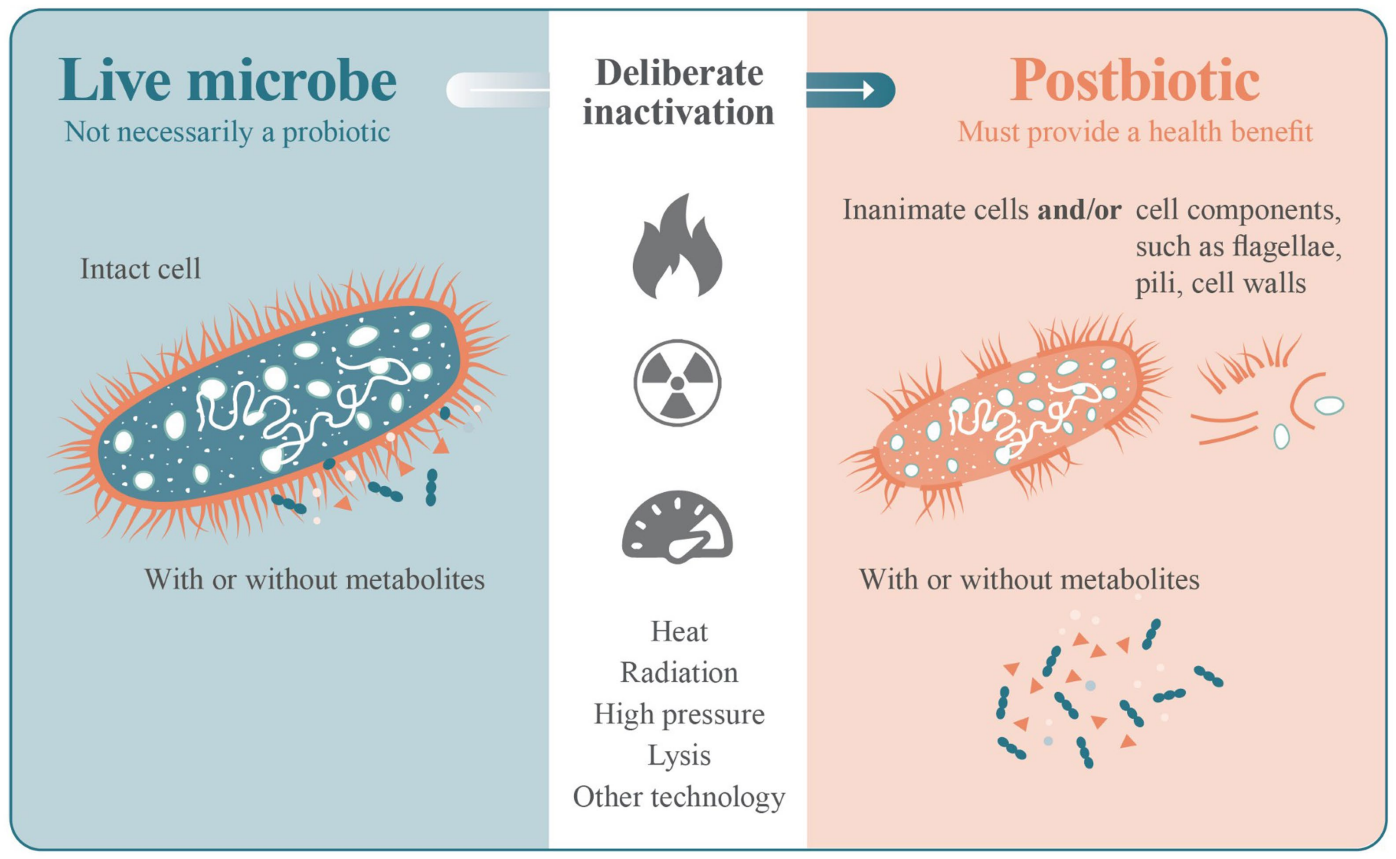
A deliberate process of viability termination (such as heat, radiation, high pressure or lysis) is applied to a live microbe as part of the manufacturing process of a postbiotic. The inactivation step may leave intact inanimate cells, cell components or a mixture of intact inanimate cells and cell components. The progenitor microbe does not necessarily have to be a probiotic.

Probiotics contain a range of cellular structures that contribute to their health benefits, and it is recognized that many of these structures retain their biological activity after cell death (Adams, 2010). The process of inactivating a live microbe and the production of a postbiotic product can also result in a mixture of potentially functional structures (Figure 1). Individually, some of these cell components have been shown to have direct effects on host physiology, with cell wall structures such as lipoteichoic acid playing a role in immunomodulation as an example (Shiraishi et al., 2016). Cellular biomass (components) can only result from living cells and are therefore ‘after-life’, and appropriate to be considered as a postbiotic.

## 4 Can metabolites be part of a postbiotic preparation?

**Figure 2:**
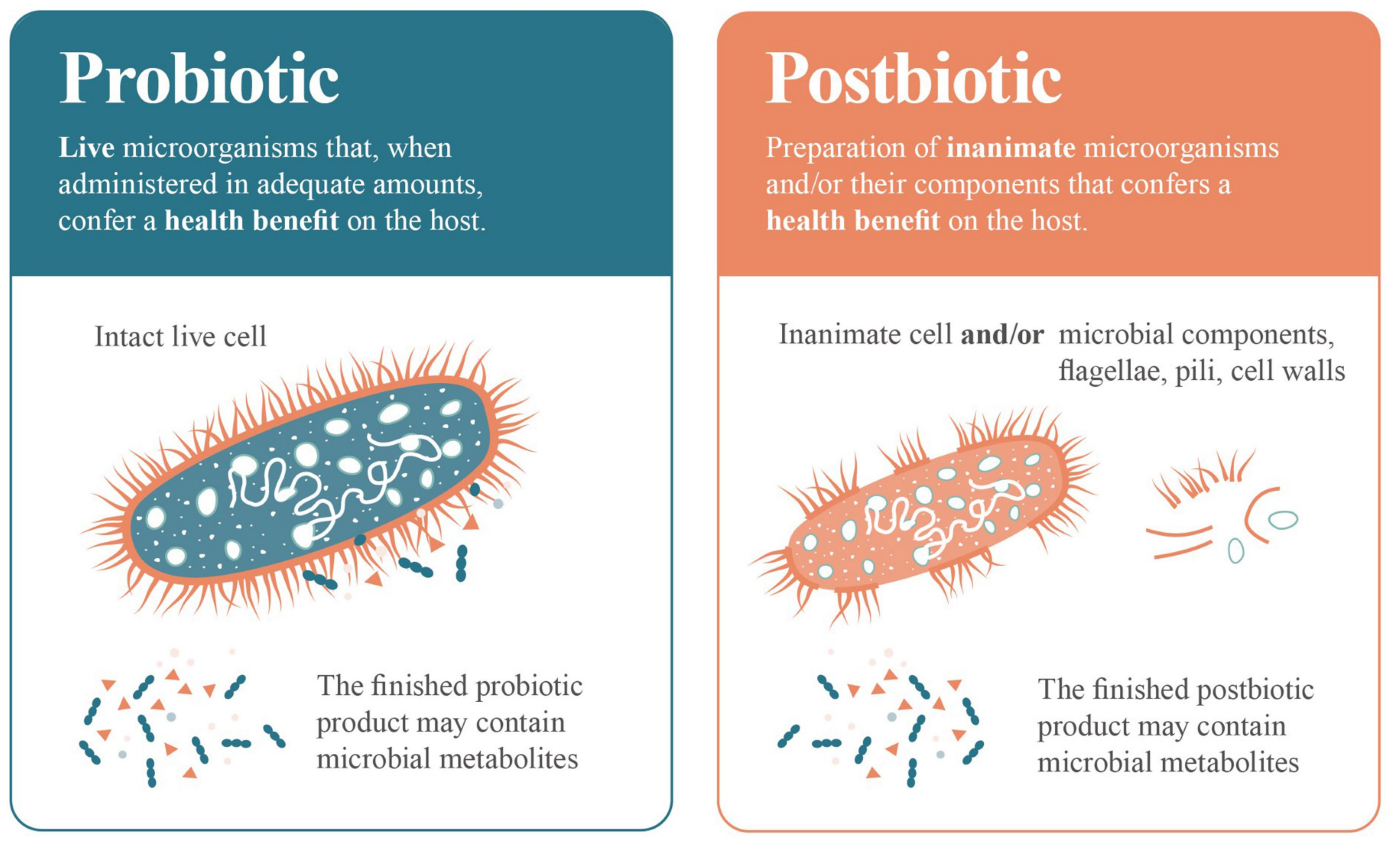
Comparing ‘probiotic’ to ‘postbiotic’. A probiotic is composed of living microbial cells that provides a health benefit. A postbiotic is composed of biologically active components (biomass such as dead cells, cell walls, surface structures, capsules) generated during the inactivation of living microbial cells. Probiotic and postbiotic products may contain or not contain metabolites.

Yes, metabolites can certainly form part of a postbiotic preparation, but they are not mandatory components of a postbiotic product. Unless inanimate microbial cells or microbial biomass is thoroughly washed and concentrated, which is not a common practice in the industry, the resulting preparation will inevitably contain some metabolites. In a similar vein, some probiotic products may also contain metabolites (Figure 2), which could play a role in any resulting health benefit.

However, if metabolites are purified or processed from inactivated cells in such a manner that no cell biomass or components remain, then they cease to be postbiotics, regardless of any health benefit. This may seem to be semantics, but definitions must delineate boundaries if they are to be useful, and the ISAPP boundary was agreed by consensus to exclude metabolites (in the absence of biomass) for the reasons stated earlier. The definition is clear that a bacterial metabolite or a mix of metabolites does not comply with the definition of a postbiotic. Further, this approach avoids the untenable situation where a preparation of one or more purified metabolites from a microbial source, assuming sufficient evidence of a health benefit, can be termed a postbiotic, yet a chemically identical preparation that is not microbially derived cannot.

## 5 What are “components” within the definition?

The term “components” refers to cellular biomass in the form of cell fragments or disrupted cells. The word components is used to recognize that microbes are composed of various large molecular weight structures and sub-structures, such as microbial cell wall compounds, cell membrane lipids, peptidoglycans and teichoic acids. Many of these cellular components are known to have immunogenic effects and could play an important role in delivering health benefits. Microbial metabolites, on the other hand, are substances produced by microbes that may be found within the cell or may be excreted and therefore external to the cell. Metabolites would be used to describe compounds such as short chain fatty acids, vitamins, or bacteriocins.

## 6 Can growth media or other ingredients be present within a postbiotic preparation?

In a practical production setting, growth media components may remain in a preparation of postbiotics after cells are harvested and inactivated. This is common to probiotics, synbiotics and postbiotics. This is one reason why it is important that the production process is controlled sufficiently to enable reproducibility of the preparation batch to batch.

## 7 Why was the term “postbiotic” chosen over other potential terms?

Terms that refer to inactivated microorganisms that confer some type of benefit had been published previously to the ISAPP definition. Such terms included ‘heat-killed probiotics,’ ‘heat-treated probiotics,’ ‘heat-inactivated probiotics,’ ‘tyndallized probiotics,’ ‘ghost probiotics’ or ‘paraprobiotics.’ One concern for all of these terms was the inclusion of the word probiotic. This implies that these inactivated microbes or preparations had to be produced from a progenitor strain that meets the criteria for a probiotic, including establishing a health benefit for the live microbe. Then after inactivation, a further study is needed to demonstrate a health benefit as a non-viable microorganism. This is an unnecessary burden on innovation. It is also possible that a strain might not confer a health benefit when alive (and therefore not qualify as a probiotic) but would provide a benefit in its inanimate form.

Other terms, such as cell fragments, cell lysates or cell biomass are already used for cell components and do not have the inbuilt requirement for a health benefit that is required for postbiotics.

## 8 What is it meant by the term “preparation”?

The biological activity of a postbiotic product will almost certainly be dependent on the identity of the progenitor strain/s but could also be influenced by the means of inactivation. The panel anticipated that the process applied to inactivate cells (e.g., heat, high pressure, radiation, lysis, etc.) could have an important impact on product functionality, as demonstrated by Wong and Ustunol (2006). In contrast, the 2011 definition of paraprobiotics (Thorakkattu et al., 2022) states that “*once a health benefit is demonstrated, the assignation of a product into the paraprobiotic category should not be influenced by the methods used for microbial cell inactivation*.” Furthermore, any filtration and fractionation techniques applied would also be expected to influence the composition of any specific postbiotic. The term preparation emphasizes that such production processes are critical to the identity and function of the postbiotic, while retaining a wide scope to accommodate innovation in other preparation methods and components.

## 9 Why was the term inanimate used?

According to the Oxford etymology dictionary, inanimate means “without vital force, or having lost life.” We propose that this is an appropriate and precise term to describe a postbiotic. Although inanimate is not a common term, as of August 2023 44 entries were found in PubMed for non-viable microbes, and 29 for inanimate microbes, suggesting the suitability of both terms to refer to microbes that are no longer alive. Within the definition, the term inanimate was used in an attempt to differentiate ‘inactive’ from ‘without life.’ Since a postbiotic is undoubtedly ‘active’ in terms of conferring a health benefit, the term ‘inactive’ could be misleading. For all practical purposes, ‘non-viable’ can be used as an appropriate synonym.

## 10 Does the definition of postbiotic include substances produced *in situ*?

Some authors suggested that postbiotics are metabolites produced after the beneficial gut bacteria metabolize prebiotics or probiotic components (Park et al., 2022), whereas other proposed that postbiotics are the metabolites generated by the microbiota (Thorakkattu et al., 2022). This vision of the concept of postbiotics, which which describes physiological processes *in situ*, does not accord with the nature of all other biotic substances. The scope of the postbiotic definition as an administered substance is aligned with previous definitions of the biotics family: probiotics (Hill et al., 2014), prebiotics (Gibson et al., 2017) and synbiotics (Swanson et al., 2020). Each of these substances is a characterized intervention that is applied for the purpose of eliciting a defined and studied health benefit. All postbiotics must impact the host either directly or indirectly (e.g., through the microbiome), leading to a health benefit. This impact may be due to the production of substances *in situ*, but the postbiotic itself is defined as the material that is administered, not the resulting metabolic byproducts.

## 11 Which microorganisms can be used as progenitors to postbiotics?

Any microbe could be used to generate a postbiotic, as long as the microbe/s is/are identified to the strain level, the preparation method is adequately described, and safety and efficacy of the preparation are demonstrated in properly conducted trials in the intended host. Even microbes that are considered pathogens when alive could be used to develop a postbiotic, as it is the case of a mixture of pathogen lysates that can be used as an immune booster for the respiratory tract (Kaczynska et al., 2022). The same is true within the probiotic definition, where any microbial species can be used if safety and efficacy are demonstrated.

## 12 Can a postbiotic contain a mixture of microorganisms?

A postbiotic can be prepared from a mixture of taxonomically distinct strains that are properly identified and characterized.

## 13 How can we understand the mechanism of action of a complex postbiotic product, which may contain inanimate cells, cell fragments and metabolites?

The potential mechanisms of action of postbiotics were discussed in the consensus paper (Salminen et al., 2021a). Many of these putative and established mechanisms are shared with probiotics and fermented products, and the final health benefit that is realized is likely due to a combination of complex interactions between the inanimate microbe or microbial components, any metabolites that are present, and the host. However, as is the case with all interventions, including probiotics and drugs, it is not essential that the mechanism of action is known. Furthermore, it is not necessary to distinguish the relative contribution to a health benefit of each component of the postbiotic.

A similar complexity exists in the example of a probiotic fermented milk. Little is known about the relative contribution to the health benefit of each of the respective components: the live cells, the dead cells, and the metabolites derived from fermentation. Knowing the relative contribution of each component is not a requirement of the definition of postbiotics, just as knowing the relative contribution of each of the strains within a multi-strain probiotic product is not mandatory to call it a probiotic. If distinguishing the relative contribution of individual components of a postbiotic product to a health benefit is of interest, a sophisticated experimental design could be applied, where all components are assessed individually and in all possible combinations, as could be the method for any complex, biologically derived preparation.

## 14 Does the process of inactivation need to be deliberate?

The intent of the postbiotic definition is that a deliberate step has been included to inactivate any living microbes (Figure 1). Efficacy studies would then be performed with this intentionally generated preparation. For example, consider *Bifidobacterium bifidum* MIMBb75, which was shown to alleviate irritable bowel syndrome when administered as a live microbe (Guglielmetti et al., 2011). Upon production as a postbiotic, a second clinical trial was performed to show that the same strain was also effective for the same condition when heat-inactivated (Andresen et al., 2020). These findings notwithstanding, it cannot be assumed that the *Bifidobacterium bifidum* MIMBb75 probiotic product would achieve postbiotic status when, for example, it loses cell viability over the course of shelf life. The deliberate, controlled and reproducible application of a heat inactivation treatment to a microbe is not the same process as the cellular death that occurs over a long shelf life and cannot be assumed to result in the same biologically active material. The deliberate application of a controlled and reproducible inactivation step should be part of the manufacturing process of a postbiotic product, as is establishing strain identity, purity, efficacy and all other quality control aspects of production.

## 15 Can a postbiotic product also contain live cells?

Some live cells may remain after the inactivation step in the process of producing a postbiotic, in general well below (several log orders) the number of inanimate cells.

In this context, it is interesting to note that the reverse challenge exists for probiotic products. Viability losses during manufacture and storage may leave a significant number of inanimate cells remaining in a probiotic product (Raymond and Champagne, 2015). In fact, the number of inanimate cells in a probiotic product could equal the number of live cells, as can be the case just after freeze-drying (Raymond and Champagne, 2015), or even exceed the number of live cells by a factor of 1–2 logs by the end of the shelf life, due to the death of probiotics during storage (Fiore et al., 2020).

## 16 How is a postbiotic measured or quantified?

Inanimate intact microbes can be measured by flow cytometry, which is able to distinguish live, dead and damaged cells. Measuring large molecular weight cellular components is difficult and a proxy may have to be used, such as total biomass. Metabolites that may be present can be quantified by HPLC or mass spectrometry technologies, among other techniques. ISAPP recognizes the importance of providing guidance on technical aspects of postbiotic characterization and quantification and intends to address this topic more fully in 2024 by convening a group of experts.

## 17 Should both metabolites and inanimate cells be characterized and quantified?

A postbiotic preparation should be characterized to a sufficient extent to allow for reproducibility and adequate quality control of individual batches. If a postbiotic contains metabolites, the manufacturer may decide to quantify one or more of these along with the inanimate cells and cell components. However, in order to meet the minimum criteria for a postbiotic, the progenitor microbe or microbes must be clearly identified and the process for making the postbiotic must be described sufficiently to enable reproduction. But the final product itself does not require detailed characterization to the level of quantification of all components and metabolites. This is analogous to probiotic products, which may also contain non-viable cells and residual metabolites from fermentation (Fiore et al., 2020), but where CFU alone is considered sufficient for quantification. Postbiotics can be considered adequately characterized based on the identity and number of inanimate cells and the method(s) used to produce them.

## 18 Should safety be demonstrated for an inanimate microbe?

Although in principle inanimate microorganisms may present fewer safety concerns than live microbes, which may translocate across the gut barrier in immunocompromised patients, by definition they are not inert substances and could potentially pose risks for safety. Proper safety assessment is an integral aspect of the development process of a postbiotic, and appropriate preclinical and clinical safety data should be collected, alongside characterization and screening of progenitor microbes and the resulting preparation for toxigenic elements.

## 19 What evidence is required to demonstrate a health benefit for a postbiotic product?

As for the other biotics (probiotics, prebiotics and synbiotics), any health benefit proposed must be supported by a well-designed (typically double-blind, randomized, placebo-controlled) intervention trial, in the target host at the appropriate dose.

## 20 Are there already products in the market that fit the postbiotic concept as defined by ISAPP?

There are several commercial products that deliver inanimate microorganisms, with or without cell components and metabolites, that are supported by clinical trials demonstrating their efficacy for gut, skin or respiratory tract applications. A non-exhaustive list includes a fermented infant formula containing spray-dry inactivated *Bifidobacterium breve* C50 plus *Streptococcus thermophilus* 065 (Szajewska et al., 2022), a combination of heat inactivated *Limosilactobacillus fermentum* CNCM MA65/4E-1b plus *Lactobacillus delbrueckii* subsp. *delbrueckii* CNCM MA65/4E-2z including their fermentation metabolites (Malagón-Rojas et al., 2020), a spray dried-inactivated strain of *Aspergillus oryzae* including its fermentation metabolites (Ríus et al., 2022), a heat-inactivated *Saccharomyces cerevisiae* strain (Pinheiro et al., 2017), heat-inactivated *Bifidobacterium bifidum* MIMBb75 (Andresen et al., 2020), heat-inactivated *L. paracasei* MCC1849 (Maehata et al., 2021), the lysate of *L. sakei* proBio65 (Rather et al., 2021), pasteurized *Akkermansia muciniphila* (Depommier et al., 2019), lysates of *Vitreoscilla filiformis* for skin applications (Gueniche et al., 2021) or a mixture of pathogens lysates for boosting the respiratory tract immunity (Kaczynska et al., 2022), among others.

## 21 Conclusion

ISAPP offers a useful definition of postbiotics – reflected in the definition and the scope of use as described in Salminen et al. (2021a) – that is designed to not be unduly prescriptive, that allows for innovation in product development, that is consistent with the root meaning of the term, and that aligns with substances being actively researched as well as products in the marketplace. Because of the existence of alternative terms for inactivated microbes and different definitions of postbiotic, the ISAPP definition has generated some discussion. Some key recurring questions have been addressed in this paper. The definition as worded was intended to be a concise reflection of a multifaceted concept. To fully understand what the definition encompasses, reading the full paper in which the definition was published is recommended as not all nuances in such a complex concept can be captured in a concise definition.

But even when read in context, the term and concept of postbiotics shares some of the complexities that exist also with the other biotic definitions. For example, concerns about understanding what components within a postbiotic are responsible for the health benefit have been expressed. Yet similar ambiguity exists with a multi-strain probiotic product. Although a given product may have been shown to confer a health benefit, exactly which mechanisms in which strains lead to it may be unknown. Similarly, concerns about quantification of a postbiotic have been expressed, yet commercial probiotic products likely contain significant numbers of dead cells, which may or may not be inert, and there is no sustained call to quantify those components of a probiotic product. These situations drive home an obvious point, which is that the perfect definition or the perfect description of any biotic does not exist. We convened this consensus group with the intention of bringing the field closer to uniting around an emerging concept with the goal of advancing the field by providing a common language useful for communicating research, consumer products and regulatory actions. Inevitably, questions will and should continue to be asked in this rapidly evolving field and we welcome this fruitful and stimulating ongoing debate.

## Data availability statement

The original contributions presented in the study are included in the article/supplementary material, further inquiries can be directed to the corresponding author.

## Author contributions

GV: Conceptualization, Writing – original draft, Writing – review & editing. MES: Writing – review & editing. MC: Writing – review & editing. CH: Writing – review & editing.
